# Co-ordinated overexpression of SIRT1 and STAT3 is associated with poor survival outcome in gastric cancer patients

**DOI:** 10.18632/oncotarget.14473

**Published:** 2017-01-03

**Authors:** Shu Zhang, Shuling Huang, Chao Deng, Yu Cao, Jun Yang, Guangxia Chen, Bin Zhang, Chaoqin Duan, Jiong Shi, Bo Kong, Helmut Friess, Nanyi Zhao, Chen Huang, Xiaoli Huang, Lei Wang, Xiaoping Zou

**Affiliations:** ^1^ Department of Gastroenterology, Drum Tower Hospital Affiliated to Medical School of Nanjing University, Nanjing, China; ^2^ Jiangsu Clinical Medical Center of Digestive Disease, Nanjing, China; ^3^ Department of Gastroenterology, The People's Hospital of Kaizhou District, Chongqing, China; ^4^ Department of Pathology, Drum Tower Hospital Affiliated to Medical School of Nanjing University, Nanjing, China; ^5^ Department of Gastroenterology, First People's Hospital of Xuzhou, Xuzhou, China; ^6^ Department of Surgery, Technical University of Munich (TUM), Munich, Germany; ^7^ Department of Human Oncology and Pathogenesis, Memorial Sloan-Kettering Cancer Center, New York, New York, USA; ^8^ Department of Epidemiology, Fuwai Hospital, Peking Union Medical College, Chinese Academy of Medical Science, Beijing, China; ^9^ Department of Gastroenterology, Nanjing Jiangbei People's Hospital Affiliated to Southeast University, Nanjing, China

**Keywords:** SIRT1, STAT3, gastric cancer, prognosis

## Abstract

In many gastric cancer patients, the disease is diagnosed in an advanced stage and therefore the mortality levels are high. Because there is a need to identify novel early diagnostic and prognostic biomarkers, we tested whether SIRT1 and STAT3 are good candidates. Towards this, we used patient tissues representing different stages of gastric cancer including gastric pre-cancerous lesions, early gastric cancer, and advanced gastric cancer, and probed SIRT1, STAT3 and phosphorylated STAT3 (pSTAT3) levels using immunohistochemistry. Our results revealed upregulated expression of SIRT1 in all stages of gastric cancer compared with noncancerous gastric mucosa, suggesting that high SIRT1 levels are likely involved in establishing gastric neoplasticity. However, STAT3 and pSTAT3 levels remained low until the gastric mucosa reached the tumor stage. Moreover, co-ordinated high expression of SIRT1 and STAT3 predicted poor overall survival for advanced gastric cancer patients. In addition, through analysis of gastric cancer patients from the TCGA dataset, we identified SIRT2 as an independent prognostic factor in gastric cancer patients. We postulate that SIRT1 and STAT3 are potential early diagnostic and prognostic markers of gastric cancer. Our study also shows that SIRT1 acts a gatekeeper during gastric tumorigenesis.

## INTRODUCTION

Gastric cancer is the third leading cause for cancer deaths worldwide. In China, it is the third most commonly diagnosed cancer and the second leading cause of cancer-related death [1, 2]. Since many patients are diagnosed with gastric cancer in an advanced stage, the prognosis is poor with an average 5-year survival rate of 14–25%, in spite of the use of conventional therapies such as surgery, chemotherapy, and radiotherapy [3]. Gastric cancer predominantly initiates from atrophic gastritis and undergoes a sequence of intestinal metaplasia, dysplasia followed by carcinoma [4]. The early gastric cancer (EGC) confined to the gastric mucosa or submucosa irrespective of the status of lymph node metastasis, and the gastric pre-cancerous lesion (PL), are both potentially curable by endoscopic submucosal dissection (ESD) [5]. Therefore, novel and diverse early diagnostic or prognostic markers and therapeutic targets for gastric cancer are required for efficient clinical detection of the cancer at an early stage.

Sirtuins (SIRT1-7) are a family of NAD(+)-dependent deacetylases that belong to the class III histone deacetylases and implicated in many cellular processes including metabolism, cell cycling and aging [6]. The best studied among the sirtuins is the SIRT1 (silent mating-type information regulation 2 homologue 1), a mammalian homologue of yeast Sir2 [7]. SIRT1 plays an important role in cell survival, cellular metabolism, stress response, and aging [8]. However, its role in tumorigenesis is ambiguous. High expression of SIRT1 has been recorded in many solid tumors [7, 9, 10]. SIRT1 has been shown to silence tumor suppressors such as p53 or activate tumor drivers as PTEN/PI3K/AKT pathway and thereby promote tumorigenesis [11, 12] However, many studies have demonstrated decreased expression of SIRT1 in some tumors and suggested a tumor suppressor function [13–15]. The conflicting data regarding SIRT1 has also been shown in gastric cancer studies. In a cohort of Korean patients with gastric carcinoma, SIRT1 expression was associated with shorter overall survival and relapse-free survival [7]. Conversely, in another cohort of Korean patients with gastric adenocarcinoma, SIRT1 expression was associated with enhanced survival [16]. Also, the function of SIRT1 during sequential development of gastric cancer, which is, from gastric pre-cancerous lesions (PL) to advanced gastric cancer (AGC) through early gastric cancer (EGC), is not known.

SIRT1 targets include both histones and non-histone proteins. One prominent SIRT1 target is STAT3 (signal transducer and activator of transcription 3), a transcriptional factor whose function is tightly regulated through SIRT1-mediated deacetylation of key lysine residues [17]. STAT3 remains constitutively activated in several human cancers including gastric cancer during cell proliferation, cell survival, immune evasion and inflammation [18]. Recently, SIRT1 was shown to inhibit the proliferation of gastric cancer cell lines *in vitro* by repressing STAT3 activity [19]. Therefore, since there was evidence for a prominent role for SIRT1 and STAT3 in gastric tumorigenesis, the aim of our study was to understand the interplay between SIRT1 and STAT3 during different tumorigenic stages of gastric cancer and assess their roles as diagnostic and prognostic biomarkers for early gastric cancer.

## RESULTS

### Clinicopathologic features of the patients

We analyzed 45 EGC patients (13 females and 32 males), of which 7 died before the last follow-up in December 2014. The age of the EGC patients ranged from 30 to 80 years old and 24 of them were below 60. According to the medical records, 15 patients had a smoking history, 11 patients had alcohol addiction, 11 patients had high blood pressure, 8 patients had type 2 diabetes, and 22 patients had Helicobacter Pylori (HP) infection (Table 1).

**Table 1:** Clinicopathological features of EGC patients based on SIRT1, STAT3, and pSTAT3 expression status

Patient characteristics	No. of patients	SIRT1 positive (*n* = 37)	SIRT1 negative (*n* = 8)	*P* value	STAT3 positive (*n* = 29)	STAT3 negative (*n* = 16)	*P* value	pSTAT3 positive (*n* = 14)	pSTAT3 negative (*n* = 31)	*P* value
**Age**										
**Median (58.3)**										
**Range (30–80)**										
**< 60**	24	18 (48.6%)	6 (75%)	0.335	18 (62.1%)	6 (37.5%)	0.114	9 (64.3%)	15 (48.4%)	0.322
**≥ 60**	21	19 (51.4%)	2 (25%)		11 (37.9%)	10 (62.5%)		5 (35.7%)	16 (51.6%)	
**Gender**										
**Female**	13	10 (27%)	3 (37.5%)	0.871	11 (37.9%)	2 (12.5%)	0.145	6 (42.9%)	7 (22.6%)	0.301
**Male**	32	27 (73%)	5 (62.5%)		18 (62.1%)	14 (87.5%)		8 (57.1%)	24 (77.4%)	
**Smoking status**										
**Smoker**	15	10 (27%)	5 (62.5%)	0.129	9 (31%)	6 (37.5%)	0.660	3 (21.4%)	12 (38.7%)	0.425
**Non-smoker**	30	27 (73%)	3 (37.5%)		20 (69%)	10 (62.5%)		11 (78.6%)	19 (61.3%)	
**Alcohol intake**										
**Yes**	11	8 (21.6%)	3 (37.5%)	0.621	8 (27.6%)	3 (18.8%)	0.766	3 (21.4%)	8 (25.8%)	1.000
**No**	34	29 (78.4%)	5 (62.5%)		21 (72.4%)	13 (81.2%)		11 (78.6%)	23 (74.2%)	
**High blood pressure**										
**Yes**	11	9 (24.3%)	2 (25%)	1.000	5 (17.2%)	6 (37.5%)	0.250	3 (21.4%)	8 (25.8%)	1.000
**No**	34	28 (75.7%)	6 (75%)		24 (82.8%)	10 (62.5%)		11 (78.6%)	23 (74.2%)	
**Diabetes**										
**Yes**	8	6 (16.2%)	2 (25%)	0.937	3 (10.3%)	5 (31.3%)	0.177	3 (21.4%)	5 (16.1%)	0.993
**No**	37	31 (83.8%)	6 (75%)		26 (89.7%)	11 (68.7%)		11 (78.6%)	26 (83.9%)	
**HP infection**										
**Yes**	22	17 (45.9%)	5 (62.5%)	0.646	12 (41.4%)	10 (62.5%)	0.175	7 (50.0%)	15 (48.4%)	0.920
**No**	23	20 (54.1%)	3 (37.5%)		17 (58.6%)	6 (37.5%)		7 (50.0%)	16 (51.6%)	
**Overall Survival**										
**Live**	38	30 (81.1%)	8	0.321	23 (79.3%)	15 (93.8%)	0.395	11 (78.6%)	27 (87.1%)	0.775
**Death**	7	7 (18.9%)	0		6 (20.7%)	1 (6.2%)		3 (21.4%)	4 (12.9%)	

Among the 83 AGC patients (30 females and 53 males) we analyzed, 33 died before the last follow-up. The age of the patients ranged from 28 to 85 years old and 36 of them were below 60. According to medical records, 15 patients had a smoking history, 19 patients had alcohol addiction, 17 patients had high blood pressure and 10 patients had type 2 diabetes. Also, 32 of the 83 AGC patients had lymph node metastasis. Based on the criteria set by the Union for International Cancer Control (UICC) TNM Classification of Malignant Tumors 7th edition, 20 AGC patients were in stage I, 21 of them in stage II, 30 of them in stage III and 12 of them in stage IV (Table 2).

**Table 2:** Clinicopathological features of AGC patients based on SIRT1, STAT3 and pSTAT3 expression status

Patient characteristics	No. of patients	SIRT1 positive (*n* = 68)	SIRT1 negative (*n* = 15)	*P* value	STAT3 positive (*n* = 52)	STAT3 negative (*n* = 31)	*P* value	pSTAT3 positive (*n* = 31)	pSTAT3 negative (*n* = 52)	*P* value
**Age**										
**Median (62)**										
**Range (28–85)**										
**< 60**	36	29 (42.6%)	7 (46.7%)	0.776	23 (44.2%)	13 (41.9%)	0.838	12 (38.7%)	24 (46.2%)	0.508
**≥ 60**	47	39 (57.4%)	8 (53.3%)		29 (55.8%)	18 (58.1%)		19 (61.3%)	28 (53.8%)	
**Gender**										
**Female**	30	25 (36.8%)	5 (33.3%)	0.802	16 (30.8%)	14 (45.2%)	0.187	10 (32.3%)	20 (38.5%)	0.569
**Male**	53	43 (63.2%)	10 (66.7%)		36 (69.2%)	17 (54.8%)		21 (67.7%)	32 (61.5%)	
**Smoking status**										
**Smoker**	15	11 (16.2%)	4 (26.7%)	0.559	9 (17.3%)	6 (19.4%)	0.815	6 (19.4%)	9 (17.3%)	0.815
**Non-smoker**	68	57 (83.8%)	11 (73.3%)		43 (82.7%)	25 (80.6%)		25 (80.6%)	43 (82.7%)	
**Alcohol intake**										
**Yes**	19	14 (20.6%)	5 (33.3%)	0.469	13 (25%)	6 (19.4%)	0.554	7 (22.6%)	12 (23.1%)	0.958
**No**	64	54 (79.4%)	10 (66.7%)		39 (75%)	25 (80.6%)		24 (77.4%)	40 (76.9%)	
**High blood pressure**										
**Yes**	17	15 (22.1%)	2 (13.3%)	0.686	14 (26.9%)	3 (9.7%)	0.060	6 (19.4%)	11 (21.2%)	0.844
**No**	66	53 (77.9%)	13 (86.7%)		38 (73.1%)	28 (90.3%)		25 (80.6%)	41 (78.8%)	
**Diabetes**										
**Yes**	10	8 (11.8%)	2 (13.3%)	1.000	6 (11.5%)	4 (12.9%)	1.000	4 (12.9%)	6 (11.5%)	1.000
**No**	73	60 (88.2%)	13 (86.7%)		46 (88.5%)	27 (87.1%)		27 (87.1%)	46 (88.5%)	
**Diameter**										
**< 5 cm**	43	35 (51.5%)	8 (53.3%)	0.896	24 (46.2%)	19 (61.3%)	0.182	15 (48.4%)	28 (53.8%)	0.630
**≥ 5 cm**	40	33 (48.5%)	7 (46.7%)		28 (53.8%)	12 (38.7%)		16 (51.6%)	24 (46.2%)	
**TNM stage**										
**I**	20	16 (23.6%)	4 (26.7%)	0.113	10 (19.2%)	10 (32.3%)	0.028*	5 (16.1%)	15 (28.8%)	0.111
**II**	21	13 (19.1%)	8 (53.3%)		11 (21.2%)	10 (32.3%)		8 (25.8%)	13 (25%)	
**III**	30	29 (42.6%)	1 (6.7%)		21 (40.4%)	9 (29%)		11 (35.5%)	19 (36.5%)	
**IV**	12	10 (14.7%)	2 (13.3%)		10 (19.2%)	2 (6.5%)		7 (22.6%)	5 (9.6%)	
**Lymph node metastasis**										
**Absent**	32	22 (32.4%)	10 (66.7%)	0.013*	16 (30.8%)	16 (51.6%)	0.059	9 (29%)	23 (44.2%)	0.169
**Present**	51	46 (67.6%)	5 (33.3%)		36 (69.2%)	15 (48.4%)		22 (71%)	29 (55.8%)	
**Overall Survival**										
**Live**	50	40 (58.8%)	10 (66.7%)	0.574	26 (50%)	24 (77.4%)	0.014*	13 (41.9%)	37 (71.2%)	0.009*
**Death**	33	28 (41.2%)	5 (33.3%)		26 (50%)	7 (22.6%)		18 (58.1%)	15 (28.8%)	

### Differential expression of SIRT1, STAT3, and pSTAT3 during different stages of gastric cancer

**Figure 1:**
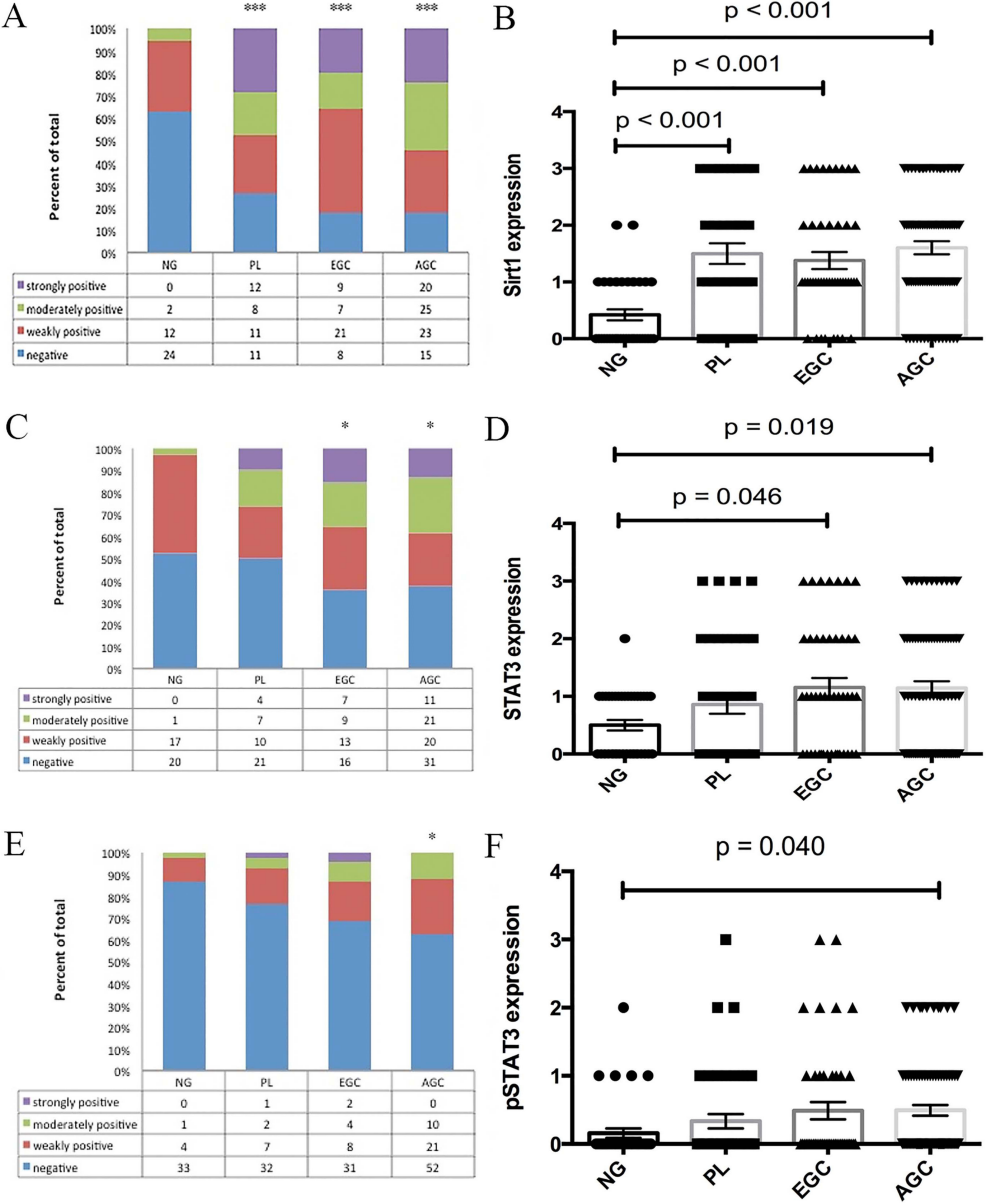
Differential expression levels of SIRT1 (**A**, **B**), STAT3 (**C**, **D**), and pSTAT3 (**E**, **F**) in patients with advanced gastric cancer, early gastric cancer, and gastric precancerous lesions compared with normal gastric mucosa. (Mean ± SEM, *p < 0.05, ***p < 0.001).

Predominantly, the SIRT1, STAT3, and pSTAT3 proteins were detected in the nucleus of gastric cancer tissues and rarely in the cytoplasm. Among the 38 control non-cancerous gastric mucosal (NG) samples, 36.8% cases stained weakly or moderately positive for SIRT1, whereas 47.4% and 13.2% stained weakly or moderately for STAT3 and pSTAT3 respectively (Supplementary Table 1). However, no strong expression of SIRT1, STAT3, or pSTAT3 was observed in any of the non-cancerous gastric mucosal specimen. In comparison, of the 45 EGC patient specimens, 82.2% were positive for SIRT1 whereas 64.4% and 31.1% were positive for STAT3 and pSTAT3, respectively (Supplementary Table 1). Similarly, among the 83 AGC patient samples, 81.9% were positive for SIRT1, whereas 62.7% and 37.3% were positive for STAT3 and pSTAT3, respectively (Supplementary Table 1). Also, among the 42 PL patient samples, 73.8% were positive for SIRT1, whereas 50% and 23.8% were positive for STAT3 and pSTAT3, respectively (Supplementary Table 1). Notably, although in comparison to control NG mucosa, SIRT1 expression was significantly upregulated in PL (*p* < 0.001), EGC (*p* < 0.001), and AGC (*p* < 0.001) as shown in Figure 1A–1B, the SIRT1 expression was comparative on comparing the PL, EGC and AGC samples. On the other hand, the expression levels of STAT3 were significantly higher in EGC (*p* = 0.046) and AGC tissues (*p* = 0.019) compared to NG tissues, whereas, there was no significant difference between PL and NG in regard to STAT3 expression (Figure 1C–1D). Further, in regard to pSTAT3, increased expression was observed in AGC compared to NG (*p* = 0.040, Figure 1E–1F).

### Correlation between the expression of SIRT1, STAT3, pSTAT3 and clinicopathologic features of early and advanced gastric cancer patients

Upon analysis, we found no correlation between the expression levels of SIRT1, STAT3 and pSTAT3 proteins and the clinicopathologic features of EGC patients (Table 1). Similarly, although SIRT1 expression and lymph node metastasis in AGC patients showed significant correlation (*p* = 0.013), there was no relationship between SIRT1 expression and the overall survival outcome (Table 2). However, the expression of STAT3 protein showed significant correlation with the TNM stage of AGC patients (*p* = 0.028; Table 2). Furthermore, the expression levels of both STAT3 and pSTAT3 correlated with the survival outcome (*p* = 0.014 and *p* = 0.009, respectively), suggesting that patients with positive STAT3 or pSTAT3 expression had a shorter overall survival time than those with negative expression.

We further classified the patients based on high (score 3) or low (scores 0, 1 and 2) expression of the protein markers and compared the relationship with the clinicopathologic features of EGC and AGC patients. We found that stronger SIRT1 expression was associated with poor overall survival than the lower SIRT1 expression in both EGC and AGC patients (*p* = 0.031 and *p* = 0.008; Supplementary Tables 2 and 3).

**Table 3:** Clinicopathologic factors and their effect on overall survival of patients with early gastric cancer as determined by univariate Cox proportional hazards regression analysis

Characteristics		No. of patients	HR (95% CI)	*P* value
SIRT1	negative	8	/	
	positive	37	/	/
SIRT1	low	36	1	
	high	9	7.592 (1.682–34.260)	0.008*
STAT3	negative	16	1	
	positive	29	3.592 (0.432–29.853)	0.237
STAT3	low	38	1	
	high	7	10.201 (2.255–46.151)	0.003*
pSTAT3	negative	31	1	
	positive	14	1.861 (0.416–8.329)	0.417
pSTAT3	low	39	1	
	high	6	2.874 (0.556–14.854)	0.208
Gender	Male	32	1	
	Female	13	1.050 (0.204–5.412)	0.954
Age	< 60	24	1	
	≥ 60	21	7.507 (0.902–62.461)	0.062
Smoking	No	30	1	
	Yes	15	0.765 (0.148–3.943)	0.749
Alcohol	No	34	1	
	Yes	11	0.803 (0.236–2.727)	0.725
High blood pressure	No	34	1	
	Yes	11	0.493 (0.059–4.097)	0.513
Diabetes	No	37	1	
	Yes	8	0.805 (0.097–6.691)	0.841
HP infection	No	23	1	
	Yes	22	1.477 (0.330–6.602)	0.610

### High expression of SIRT1 and STAT3 leads to poor survival outcomes of early and advanced gastric cancer patients

**Figure 2:**
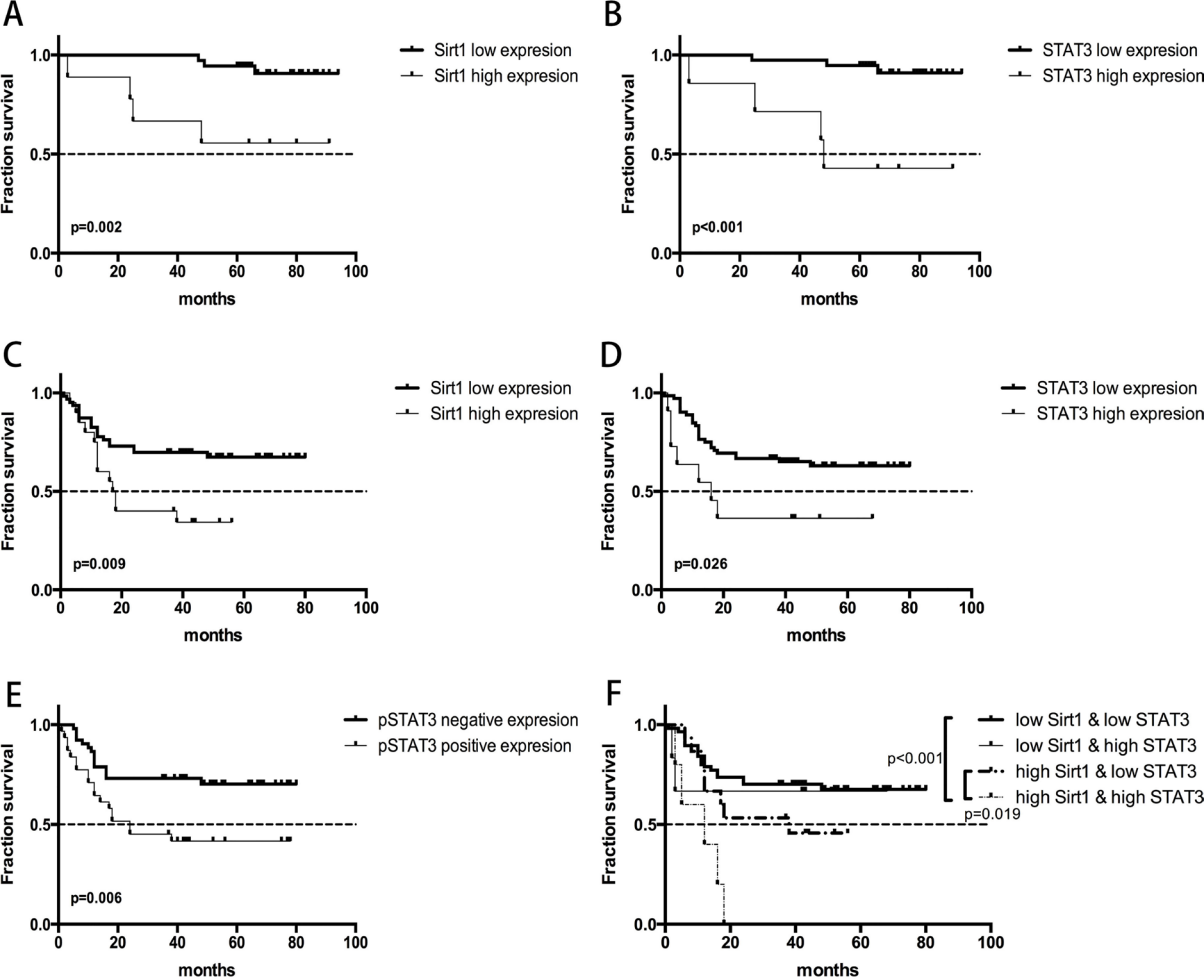
Overall survival curves demonstrating the relationship between survival prognosis of gastric cancer patients and gene expression of SIRT1 and STAT3 (**A**) High versus low SIRT1 expression in early gastric cancer patients; (**B**) High versus low STAT3 expression in early gastric cancer patients; (**C**) High versus low SIRT1 expression in advanced gastric cancer patients; (**D**) High versus low STAT3 expression in advanced gastric cancer patients; (**E**) Positive versus negative pSTAT3 expression in early gastric cancer patients; (**F**) Combined effects of SIRT1 and STAT3 expression in predicting survival outcomes of advanced gastric cancer patients.

We investigated the relationship between SIRT1 expression levels and the overall survival of gastric cancer patients (Figure 4A and 4D). The Kaplan-Meier survival analysis revealed that the low SIRT1 expression group survived longer (90.39 months) than the high SIRT1expression group (61.67 months) among EGC patients (*p* = 0.002; Figure 2A). Similar finding was observed in AGC patients (*p* = 0.009), with the low SIRT1 expression group exhibiting longer overall survival (58.18 months) than the higher SIRT1 expression group (28.27 months) as shown in Figure 2C.

To investigate the link between STAT3 expression levels and overall survival of patients, we divided the patients into STAT3-high and -low expression groups (Figure 4C and 4F). Our analysis demonstrated that low STAT3 expressing EGC patients survived longer (89.91 months) than those expressing high STAT3 levels (56.67 months; *p* < 0.001) as shown in Figure 2B. Similarly, AGC patients with low STAT3 expression showed longer overall survival time (55.79 months) than those with high STAT3 expression (30.09 months; *p* = 0.026) as shown in Figure 2D.

In conjunction to the above findings, we observed that low SIRT1 and STAT3 expressing AGC patients displayed significant progression free survival (PFS) benefits compared to high SIRT1 (*p* = 0.004) and STAT3 (*p* = 0.014) expressing AGC patients (Supplementary Figures 1 and 2).

To determine if the combinatorial expression of SIRT1 and STAT3 could predict survival outcomes, the AGC patients were further divided into 4 groups as follows: (1) patients with low SIRT1 expression & low STAT3 expression; (2) patients with low SIRT1 expression & high STAT3 expression; (3) patients with high SIRT1 expression & low STAT3 expression and (4) patients with high SIRT1 expression & high STAT3 expression. Since all the five patients in group 4 had died before the last follow-up, we concluded that group 4 (high SIRT1 and high STAT3) had the most significantly decreased overall survival rate compared to groups 1 and 3 (*p* < 0.001 and *p* = 0.019, respectively; Figure 2F).

Following these findings, we performed the univariate Cox proportional hazard analysis to relate the expression level of each protein, the clinicopathologic features and the overall survival outcome of the EGC and the AGC patients (Tables 3 and 4). The overall survival time was significantly shorter for AGC patients demonstrating high TNM stage (Stage IV), large tumor (> 5 cm in diameter) and presence of lymph node metastasis (Table 4). In addition, the univariate Cox regression model validated the prolonged survival of low SIRT1 and STAT3 expressing EGC and AGC patients.

**Table 4:** Clinicopathologic factors and their effect on overall survival of patients with advanced gastric cancer as determined by univariate cox proportional hazards regression analysis

Characteristics		No. of patients	HR (95% CI)	*P* value
SIRT1	negative	15	1	
	positive	68	1.200 (0.463–3.109)	0.708
SIRT1	low	62	1	
	high	20	2.433 (1.205–4.912)	0.013*
STAT3	negative	31	1	
	positive	52	2.718 (1.179–6.270)	0.019*
STAT3	low	72	1	
	high	11	2.469 (1.070–5.700)	0.034*
pSTAT3	negative	52	1	
	positive	31	2.491 (1.253–4.952)	0.009*
pSTAT3	low	73	1	
	high	10	1.449 (0.559–3.753)	0.445
Gender	Male	53	1	
	Female	30	0.766 (0.364–1.610)	0.481
Age	< 60	36	1	
	≥ 60	47	1.968 (0.935–4.141)	0.074
TNM stage	I/II/III	71	1	
	IV	12	13.860 (6.301–30.486)	< 0.001*
Diameter	< 5 cm	43	1	
	≥ 5 cm	40	3.665 (1.737–7.736)	0.001*
Lymph node metastasis	Absent	32	1	
	Present	51	6.173 (2.164–17.604)	0.001*
Smoking	No	68	1	
	Yes	15	1.287 (0.558–2.968)	0.554
Alcohol	No	64	1	
	Yes	19	1.287 (0.598–2.770)	0.519
High blood pressure	No	66	1	
	Yes	17	2.169 (1.050–4.481)	0.036
Diabetes	No	73	1	
	Yes	10	1.313 (0.507–3.402)	0.575

Multivariate Cox proportional hazard analysis was also performed to evaluate the relationship of each protein to various clinicopathologic features by considering high or positive expression for the included proteins separately because the thresholds for the proteins were heterogenous resulting in varied correlations between levels of SIRT1 andSTAT3 proteins with the survival outcomes of the gastric cancer patients. Since the staining for pSTAT3 protein was generally weak to moderate, we did not set up a threshold as high vs. low for pSTAT3. We then adjusted a series of factors that included gender, age, smoking history, alcohol addiction, high blood pressure, type 2 diabetes and HP infection. We observed that in EGC patients, high SIRT1 expression exhibited poor overall survival outcomes compared to low SIRT1 expression (HR = 6.664, 95% CI 1.359 to 32.688; *p* = 0.019). Similar findings were noted for EGC patients with high STAT3 expression (HR = 9.065, 95% CI 1.869 to 43.956; *p* = 0.006). We included additional factors like TNM stage, tumor size and status of lymph node metastasis while performing the multivariate regression model for AGC patients. Our analysis revealed that high STAT3 expression (HR = 2.847, 95% CI 1.142 to 7.097; *p* = 0.025) and positive pSTAT3 expression (HR = 2.326, 95% CI 1.141 to 4.742; *p* = 0.020) indicated poor overall survival for AGC patients. Also, the other factors namely, TNM stage, tumor size, and diabetes functioned as independent prognostic factors for AGC patients (*p* < 0.001, *p* < 0.001, and *p* = 0.040, respectively) as shown in Table 5.

**Table 5:** Clinicopathologic factors and their effect on overall survival of patients with advanced gastric cancer as determined by multivariate Cox proportional hazards regression analysis

Characteristics		No. of patients	HR (95% CI)	*P* value
STAT3	low	72	1	
	high	11	2.847 (1.142–7.097)	0.025*
pSTAT3	negative	52	1	
	positive	31	2.326 (1.141–4.742)	0.020*
TNM stage	I/II/III	71	1	
	IV	12	17.481 (6.821–44.796)	< 0.001*
Diameter	< 5 cm	43	1	
	≥ 5 cm	40	4.456 (1.980–10.029)	< 0.001*
Diabetes	No	73	1	
	Yes	10	2.985 (1.054–8.454)	0.040*

### Upregulation of SIRT1 is associated with STAT3 activation in EGC patients

To determine the correlation between SIRT1 expression and STAT3 activity during gastric cancer development, we performed the Spearman's correlation analysis in all tissues including the NG mucosa, PL, EGC and AGC tissues. We observed a positive correlation between the expression of SIRT1 and STAT3 (*p* = 0.046) as well as the expression of SIRT1 and pSTAT3 (*p* = 0.010) in EGC patients. However, there was no correlation between SIRT1 and STAT3/pSTAT3 expression in the NG, PL and AGC tissues. Therefore, we postulated that high SIRT1 expression was positively correlated with STAT3 expression and its activation in EGC patients.

### High expression of SIRT2 is associated with poor survival benefit for patients with gastric cancer from the TCGA dataset

**Figure 3:**
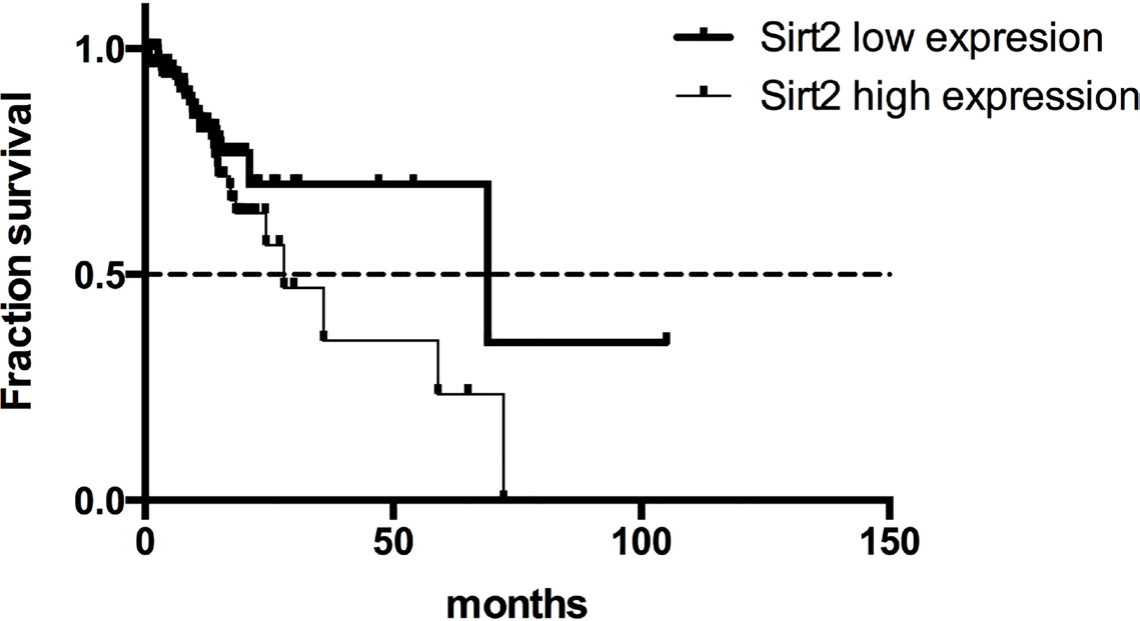
Overall survival curves for gastric cancer patients from the TCGA dataset with high or low SIRT2 expression

We further validated our findings by analyzing the TCGA gastric cancer dataset with 196 patients. We extracted mRNA expression values for STAT3 and all the Sirtuins family members (SIRT1-7) from the RNA-seq data in the cBioportal. Though no significant association was observed between gene expression and survival outcomes of the patients through univariate regression analysis (Supplementary Table 4), high expression of SIRT2 was associated with increased overall mortality (HR = 2.123, 95% CI 1.089 to 4.137; *p* = 0.027) after adjusting for gender, age, lymph node metastasis, TNM stage, EBV presence, total number of mutations and presence of common mutations (TP53, ARID1A, PIK3CA, RHOA, and KRAS) as shown in Supplementary Table 5. Kaplan-Meier survival analysis demonstrated that the SIRT2-low expression group had a longer overall survival time (62.00 months) than the SIRT2-high expression group (27.93 months; Figure 3). Also, the multivariate regression analysis showed that high expression of SIRT2 resulted in poor PFS (142 patients included; HR = 3.509, 95% CI 1.246 to 9.878, *p* = 0.017; Supplementary Table 6). However, there was no correlation between SIRT1/STAT3 expression and survival outcome of the patients based on multivariate regression analysis.

**Figure 4:**
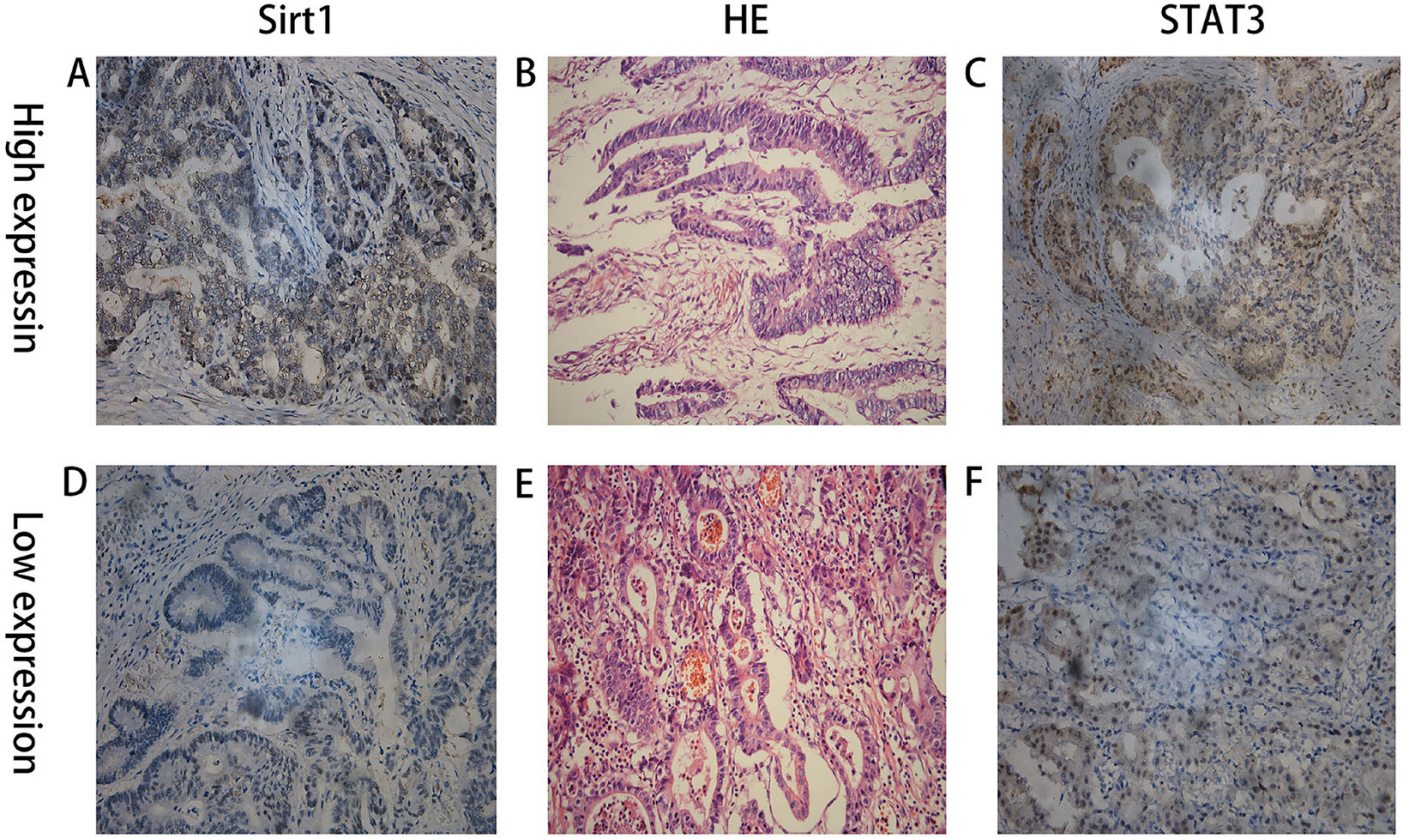
IHC staining showing high and low expression of SIRT1 and STAT3 in two patients with advanced gastric cancer and their matched HE staining slides (**A**) High expression of SIRT1; (**B**) the matched HE staining slide of A & C; (**C**) High expression of STAT3; (A), (B), and (C) were from the same patient; (**D**) low expression of SIRT1; (**E**) the matched HE staining slide of D & F; (**F**) low expression of STAT3; (D), (E), and (F) were from the same patient.

### Differential expression of SIRT1, SIRT2, and STAT3 between gastric cancer and paired normal gastric mucosa in gastric cancer patients from 2 GEO datasets

Based on RNA-seq data, we extracted the mRNA expression values of SIRT1, SIRT2 and STAT3 from the GSE63089 and GSE29272 datasets using the GEO2R platform. Expression of SIRT1 and STAT3 was significantly higher in the gastric cancer tissues than in the paired normal gastric mucosa for patients in both the GSE63089 (*p* < 0.001 for both SIRT1 and STAT3) and GSE29272 (*p* = 0.003 for SIRT1 and *p* < 0.001 STAT3) as shown in Supplementary Figures 3–6. In the GSE63089 dataset, we observed increased SIRT2 expression for gastric cancer tissues than paired normal tissues (*p* = 0.001; Supplementary Figure 7). However, we surprisingly observed that expression of SIRT2 was significantly downregulated in gastric cancer tissues in patients from GSE29272 (Supplementary Figure 8). Given these confounding findings, the SIRT2 function in gastric tumorigenesis needs to be thoroughly investigated in the future.

## DISCUSSION

Many countries face a severe burden of high incidence and mortality due to gastric cancer. Improvements in the endoscopic approaches have enabled the cure of early gastric cancer and gastric precancerous lesions due to high resection and low recurrence rates. Yet, further effective early diagnostic and prognostic biomarkers need to be identified to guide clinical therapy.

Many studies have shown contradictory roles for SIRT1 either promoting cancer cell survival or accelerating cell death in a variety of cancer types. Whereas a cohort of Chinese patients with gastric cancer showed significantly higher SIRT1 expression in tumor tissues than in normal gastric mucosa [20], another cohort of Chinese patients showed that SIRT1 was downregulated [13]. Besides, contradicting associations between SIRT1 expression and survival outcomes were observed in two different cohorts of Korean gastric cancer patients [7, 16]. These findings created difficulty in predicting the prognostic effect of SIRT1 in gastric cancer patients. Therefore, in this study we studied SIRT1 expression in our own cohort of patients as well as patients from public datasets to clarify the role of SIRT1 in gastric cancer. We also studied STAT3, a transcriptional factor in tandem with SIRT1 because STAT3 is an important substrate of SIRT1 and SIRT1 has been reported to suppress the inhibitory effects of STAT3 on gluconeogenesis [17]. STAT3 has also been shown to accelerate tumorigenesis by upregulating cell survival proteins, downregulating tumor suppressors and acquiring drug resistance [21]. Therefore, we studied if SIRT1 and STAT3 collaborate during gastric tumor progression.

Our data showed significant upregulation of SIRT1 in all the three gastric cancer stages (PL, EGC and AGC) in comparison to non-cancerous gastric tissue. However, no significant differences of SIRT1 expression were observed among the three cancer stages suggesting that SIRT1 protein expression was upregulated during early gastric neoplastic development and then maintained. However, the expression of STAT3 and pSTAT3 proteins remained low until the gastric mucosa reached the tumor stage. Also, as expected, the expression levels of SIRT1 and STAT3 mRNA were significantly higher in gastric cancer tissues than in matched normal mucosa from public datasets.

Further, we showed that positive SIRT1 expression was significantly associated with lymph node metastasis in AGC patients indicating that SIRT1 expression lead to tumor metastasis that is consistent with the previous report in a cohort of Korean patients [7]. However, there was no correlation between SIRT1 expression and the overall survival of patients. Also, STAT3 expression was closely related to higher TNM stage in the AGC patients and both STAT3 and pSTAT3 expression were associated with poor overall survival outcomes.

To further substantiate the correlation between SIRT1 expression and survival outcome of patients with gastric cancer, we divided the patients into high and low SIRT1 and STAT3 expression groups. Univariate Cox proportional hazard analysis revealed a 2.4-fold increase in mortality for AGC patients with high SIRT1 expression compared to low SIRT1 expression and a 2.5-fold increase in mortality for patients with high STAT3 expression. There was a 7.6 fold increase in mortality among the high SIRT1 expressing EGC patients group, whereas, a 10.2 fold increase in mortality was noted among the high STAT3 expression category. Furthermore, multivariate Cox proportional hazard analysis demonstrated that both the high SIRT1 and STAT3 expression levels functioned as independent prognostic factors in EGC patients. For the AGC patients, high expression of STAT3, positive expression of pSTAT3, TNM stage, tumor size, and type 2- diabetes were all categorized as independent prognostic markers.

Only few studies have explored the counteracting effects of SIRT1 and STAT3 in tumorigenesis. Of these, one study showed contrasting data that SIRT1 inhibited STAT3 activity in gastric cancer cell lines *in vitro* [19]. Our study was the first to investigate the collaborating effects of SIRT1 and STAT3 in gastric cancer patients. In our cohort of EGC patients, SIRT1 expression was positively correlated with STAT3 and pSTAT3 expression indicating that upregulation of SIRT1 expression was associated with cellular STAT3 activation. The underlying mechanism for this process was not explored in this study. However previous studies have shown that SIRT1 inhibited the phosphorylation and function of STAT3 by deacetylating key STAT3 lysine sites in murine liver cells [17]. Therefore, we hypothesized that STAT3 may not be a direct target of SIRT1 in our cohort of gastric cancer patients. The possible hypothesis was that SIRT1 dysregulated the transcriptional activity of its downstream targets that lead to the activation of STAT3. The lack of association between SIRT1 and STAT3 expression in AGC patients supported independent functionality of SIRT1 and STAT3 in promoting gastric tumorigenesis after malignancy was attained. Further, we found that combinatorial high expression of both SIRT1 and STAT3 predicted poor survival outcomes in AGC patients compared to those with low expression of at least one of the two proteins. This supported the hypothesis that SIRT1 and STAT3 functioned independently in advanced tumors as important oncogenes to maintain the malignancy of stomach tumors resulting in poor survival outcomes in patients with high expression of both SIRT1 and STAT3.

Another interesting finding in our study was that although SIRT1 expression was increased very early during gastric tumorigenesis, only strong expression of SIRT1 predicted poor survival outcomes for both the EGC and AGC patients. This suggests that SIRT1 is a gatekeeper for the normal state of gastric mucosa. Once precancerous lesions have occurred, SIRT1 signaling is elevated to protect the lesions from progressing rapidly. However SIRT1 expression must be maintained below a critical threshold. When this threshold is achieved, higher SIRT1 expression leads to increased overall mortality of the patients possibly by promoting metastasis. In conclusion, the balanced SIRT1 signaling keeps the gastric mucosa stay in normal status, and SIRT1 functions more like an oncogenic maintainer rather than an oncogenic driver. Alone, SIRT1 expression is limited in initiating gastric tumorigenesis. However, in combination with other stimulating factors, it assists in development of gastric neoplasia and subsequently when its expression reaches a high threshold, SIRT1 maintains the malignancy of gastric cancer. Therefore, the various contradictory reports on the differential SIRT1 expression between gastric cancer and normal gastric tissues probably reflect the complex association between its expression and the different stages of cancer.

However, we failed to observe close correlation between high SIRT1 and STAT3 mRNA expression and the clinical outcomes in the public datasets. There are two possible reasons: (1) The mRNA expression of SIRT1 and STAT3 may not be consistent with its protein expression level and (2) We were unable to set up appropriate threshold to subdivide the patients based on the SIRT1/STAT3 expression levels. In addition, we observed that SIRT2 mRNA expression was an independent prognostic marker for these patients. SIRT2 also plays contradictory roles in tumor development. SIRT2 overexpression suppresses cell proliferation and induces apoptosis in cancer cells. On the other hand, treatment with different SIRT2 inhibitors induces cancer cell apoptosis [22]. Additionally, aberrant SIRT2 expression was reported to predict poor survival outcome of esophageal and gastroesophageal junctional adenocarcinoma patients [23]. Based on the two GEO cohorts of patients with gastric cancer, SIRT2 mRNA expression could either be increased or decreased in tumor tissues compared with normal gastric mucosa. Therefore more studies are necessary to determine the exact function of SIRT2 in gastric tumor progression.

In conclusion, SIRT1 is a potential pathological biomarker for early diagnosis and prognosis of gastric cancer and functions as a gatekeeper during stomach tumorigenesis. SIRT1 protein expression is upregulated at a very early stage and maintained through the sequential gastric tumorigenic progression. High expression of SIRT1 protein helps maintain the malignant status of stomach tumors. Although STAT3 may not be the direct target of SIRT1 in gastric cancer, combinatorial high expression of both these proteins can predict the worst survival outcome. Therefore, individual or combinatorial inhibition of SIRT1/STAT3 accompanied with ESD or surgeries might be a promising therapy to patients with EGC or AGC.

## MATERIALS AND METHODS

### Patients and tissue samples

Tissues from PL (2010–2012), EGC (2006–2008) and AGC (2008–2011) patients with complete clinical information and available paraffin-embedded tissue blocks were enrolled in this study. Those patients who had received radiation therapy or chemotherapy before surgery or ESD operations were excluded from our analysis. 83 AGC patients in our study had undergone surgery in the General Surgery Department of Nanjing Drum Tower Hospital. 45 EGC patients who had received surgery or ESD operations were also included in our study. In addition, we had 42 patients with precancerous lesions of the stomach (low grade or high grade dysplasia) receiving ESD operations in the Gastro-endoscopy Center in our hospital. As controls, we enrolled 38 healthy volunteers to obtain noncancerous gastric (NG) mucosa through gastro-endoscopic checking in our Gastro-endoscopy Center. The protocols were approved by the Ethical Committee and Institutional Review Board of Nanjing Drum Tower Hospital of Nanjing University Medical School, and informed consent was obtained in writing from both patients and healthy volunteers. All clinicopathologic characteristics, including age, gender, tumor stage, alcohol intake, smoking history and prognostic information were retrospectively collected from the patient records. All the data collection, data analysis, and experiments were carried out in accordance with the approved protocol.

### Immunohistochemistry (IHC) of paraffin-embedded sections

Formalin-fixed and paraffin-embedded specimens were sectioned at 4-μm thickness, deparaffinized, blocked and incubated at 4°C overnight with primary antibody, followed by incubation with horseradish peroxidase-labeled secondary antibody. The slides were stained with diaminobenzidine as the color reagent followed by counterstaining with hematoxylin for nuclei. Negative controls were achieved by substituting the primary antibodies with PBS. The primary antibodies used in this study were: rabbit monoclonal antibody to SIRT1 (1:200, Abcam, UK, ab32441), mouse monoclonal antibody to STAT3 (1:500, CST, USA, #9139) and rabbit monoclonal antibody to phospho-STAT3 (Tyr705) (1:400, CST, USA, #9145).

The IHC sections were scored by two different pathologists independently. Disagreement was resolved by consensus. A third pathologist was invited to review the scores judged by the two pathologists. Each section was scored according to the intensity and percentage of positively stained cells: 0 (negative, 0–5% positive cells), 1 (weakly positive, 5–30% positive cells), 2 (moderately positive, 30–60% positive cells) and 3 (strongly positive, > 60% positive cells).

### Statistical analysis

The Sirtuins 1–7 and STAT3 mRNA expression data from The Cancer Genome Atlas (TCGA) stomach cancer datasets and the related clinicopathologic information of the included patients was obtained from the cBioPortal for Cancer Genomics generated by Memorial Sloan-Kettering Cancer Center [24, 25]. GEO datasets as GSE29272 and GSE63089 were used to compare the expression of SIRT1, SIRT2 and STAT3 between gastric cancer samples and their paired normal gastric tissues.

All the data analysis from our cohort and TCGA/GSE datasets was performed using SPSS software version 22.0 (SPSS Inc., USA). For comparisons, the Kruskal-Wallis one-way analysis of variance was performed to investigate the differential expression of each protein among all 4 groups of patients. The differences in SIRT1, STAT3 and pSTAT3 expression among patients were analyzed by *t*-test for continuous variables and chi-squared test for categorical variables. Correlations were analyzed by the Spearman Rank-Order method. Log-rank tests were performed on Kaplan-Meier survival curves to determine any significant relationships between gene expression and patient outcomes. Univariate and multivariate survival analyses were performed with the Cox proportional hazards regression model. Multivariate regression models were fitted to identify independent factors related to overall mortality and only variables with *P* < 0.1 were retained for multivariate analysis. Results were expressed as hazard ratio (HR) with 95% confident intervals (CI). All tests were two-sided and *P* < 0.05 was considered statistically significant.

## SUPPLEMENTARY MATERIALS TABLES AND FIGURES



## Abbreviations

NG: normal gastric mucosa
PL: precancerous lesions
EGC: early gastric cancer
AGC: advanced gastric cancer
ESD: endoscopic submucosal dissection
pSTAT3: phosphorylated STAT3
HP: helicobacter pylori
HR: hazard ratio
CI: confident intervals
IHC: immunohistochemistry
