# Supplementary material for: Co-ordinated overexpression of SIRT1 and STAT3 is associated with poor survival outcome in gastric cancer patients
Source: Oncotarget. 2017 Jan 3;8(12):18848–60. doi: 10.18632/oncotarget.14473 (PMC5386652; doi:10.18632/oncotarget.14473)
Supplement: Supplementary file 1 [file oncotarget-08-18848-s001.pdf]

Co-ordinated overexpression of SIRT1 and STAT3 is associated with poor survival outcome in gastric cancer patients

Supplementary Materials

Supplementary Table 1: Scoring of SIRT1, STAT3 and pSTAT3 expression levels in gastric cancer progression

Antibody	Score	NG	PL	EGC	AGC	Total
	0	24	11	8	15	58
	1	12	11	21	23	67
SIRT1	2	2	8	7	25	42
	3	0	12	9	20	41
	Total	38	42	45	83	208
	0	20	21	16	31	88
	1	17	10	13	20	60
STAT3	2	1	7	9	21	38
	3	0	4	7	11	22
	Total	38	42	45	83	208
	0	33	32	31	52	148
	1	4	7	8	21	40
pSTAT3	2	1	2	4	10	17
	3	0	1	2	0	3
	Total	38	42	45	83	208

NG, noncancerous gastric mucosa; PL, gastric precancerous lesions; EGC, early gastric cancer; AGC, advanced gastric cancer.

Supplementary Table 2: Classification of clinicopathological features of EGC patients based on SIRT1, STAT3 and pSTAT3 expression levels (high versus low)

Patient characteristics	No. of patients	SIRT1 high (*n* = 9)	SIRT1 low (*n* = 36)	*P* value	STAT3 high (*n* = 7)	STAT3 low (*n* = 38)	*P* value	pSTAT3 high (*n* = 6)	pSTAT3 low (*n* = 39)	*P* value
**Age**										
**Median (58.3)**										
**Range (30-80)**										
**< 60**	24	2 (22.2%)	22 (61.1%)	0.086	3 (42.9%)	21 (55.3%)	0.847	3 (50.0%)	21 (53.8%)	1.000
**≥ 60**	21	7 (77.8%)	14 (38.9%)		4 (57.1%)	17 (44.7%)		3 (50.0%)	18 (46.2%)	
**Gender**										
**Female**	13	2 (22.2%)	11 (30.6%)	0.934	3 (42.9%)	10 (26.3%)	0.665	4 (66.7%)	9 (23.1%)	0.087
**Male**	32	7 (77.8%)	25 (69.4%)		4 (57.1%)	28 (73.7%)		2 (33.3%)	30 (76.9%)	
**Smoking status**										
**Smoker**	15	2 (22.2%)	13 (36.1%)	0.693	2 (28.6%)	13 (34.2%)	1.000	1 (16.7%)	14 (35.9%)	0.642
**Non-smoker**	30	7 (77.8%)	23 (63.9%)		5 (71.4%)	25 (65.8%)		5 (83.3%)	25 (64.1%)	
**Alcohol intake**										
**Yes**	11	3 (33.3%)	8 (22.2%)	0.795	1 (14.3%)	10 (26.3%)	0.840	1 (16.7%)	10 (25.6%)	1.000
**No**	34	6 (66.7%)	28 (77.8%)		6 (85.7%)	28 (73.7%)		5 (83.3%)	29 (74.4%)	
**High blood pressure**										
**Yes**	11	3 (33.3%)	8 (22.2%)	0.795	1 (14.3%)	10 (26.3%)	0.840	2 (33.3%)	9 (23.1%)	0.973
**No**	34	6 (66.7%)	28 (77.8%)		6 (85.7%)	28 (73.7%)		4 (66.7%)	30 (76.9%)	
**Diabetes**										
**Yes**	8	2 (22.2%)	6 (16.7%)	1.000	1 (14.3%)	7 (18.4%)	1.000	2 (33.3%)	6 (15.4%)	0.619
**No**	37	7 (77.8%)	30 (83.3%)		6 (85.7%)	31 (81.6%)		4 (66.7%)	33 (84.6%)	
**HP infection**										
**Yes**	22	5 (55.6%)	17 (47.2%)	0.941	4 (57.1%)	18 (47.4%)	0.949	3 (50.0%)	19 (48.7%)	1.000
**No**	23	4 (44.4%)	19 (52.8%)		3 (42.9%)	20 (52.6%)		3 (50.0%)	20 (51.3%)	
**Overall Survival**										
**Live**	38	5 (55.6%)	33 (91.7%)	0.031*	3 (42.9%)	35 (92.1%)	0.006*	4 (66.7%)	34 (87.2%)	0.493
**Death**	7	4 (44.4%)	3 (8.3%)		4 (57.1%)	3 (7.9%)		2 (33.3%)	5 (12.8%)	

Supplementary Table 3: Classification of clinicopathological features of AGC patients based on SIRT1, STAT3 and pSTAT3 expression levels (high versus low). See Supplementary_Table_3

Supplementary Table 4: Effect of clinicopathological factors on overall survival of patients from TCGA stomach cancer dataset based on univariate Cox proportional hazards regression analysis

Characteristics		No. of patients	HR (95% CI)	*P* value
SIRT1	low	101	1	
	high	95	0.831 (0.445–1.550)	0.560
SIRT2	low	100	1	
	high	96	1.459 (0.790–2.694)	0.227
SIRT3	low	124	1	
	high	72	0.919 (0.495–1.709)	0.790
SIRT4	low	108	1	
	high	88	1.010 (0.548–1.863)	0.974
SIRT5	low	122	1	
	high	74	0.793 (0.415–1.515)	0.482
SIRT6	low	117	1	
	high	79	0.706 (0.376–1.326)	0.279
SIRT7	low	102	1	
	high	94	0.886 (0.487–1.614)	0.693
STAT3	low	111	1	
	high	85	1.337 (0.722–2.474)	0.356
Gender	male	127	1	
	female	72	0.892 (0.461–1.725)	0.734
Age	< 60	62	1	
	≥ 60	134	0.987 (0.510–1.908)	0.969
pT status	pT1/T2/T3	148	1	
	pT4	48	2.587 (1.317–5.080)	0.006*
Lymph node metastasis	absent	64	1	
	present	132	3.006 (1.332–6.783)	0.008*
TNM stage	I/II/III	184	1	
	IV	12	9.190 (3.922–21.539)	< 0.001*
EBV	absent	177	1	
	present	19	1.082 (0.383–3.061)	0.881
No. of mutation	< 300	151	1	
	≥ 300	45	0.548 (0.243–1.237)	0.148
TP53 mutation	absent	105	1	
	present	91	0.661 (0.353–1.237)	0.195
ARID1A mutation	absent	137	1	
	present	59	1.050 (0.553–1.992)	0.882
PIK3CA mutation	absent	157	1	
	present	39	0.965 (0.425–2.188)	0.931
RHOA mutation	absent	183	1	
	present	13	0.612 (0.147–2.551)	0.500
KRAS mutation	absent	181	1	
	present	15	1.627 (0.576–4.593)	0.358

Supplementary Table 5: Effect of clinicopathological factors on overall survival of patients from TCGA stomach cancer dataset based on multivariate cox proportional hazards regression analysis

Characteristics		No. of patients	HR (95% CI)	*P* value
SIRT2	low	100	1	
	high	96	2.123 (1.089–4.137)	0.027*
Lymph node metastasis	absent	64	1	
	present	132	2.801 (1.198–6.548)	0.017*
TNM stage	I/II/III	184	1	
	IV	12	9.930 (3.915–25.191)	< 0.001*

Supplementary Table 6: Effect of clinicopathological factors on progression-free survival of patients from TCGA stomach cancer dataset based on multivariate cox proportional hazards regression analysis

Characteristics		No. of patients	HR (95% CI)	*P* value
SIRT2	low	70	1	
	high	72	3.509 (1.246–9.878)	0.017*
Lymph node metastasis	absent	49	1	
	present	93	3.631 (1.031–12.793)	0.045*
TNM stage	I/II/III	138	1	
	IV	4	17.823 (4.296–73.952)	< 0.001*

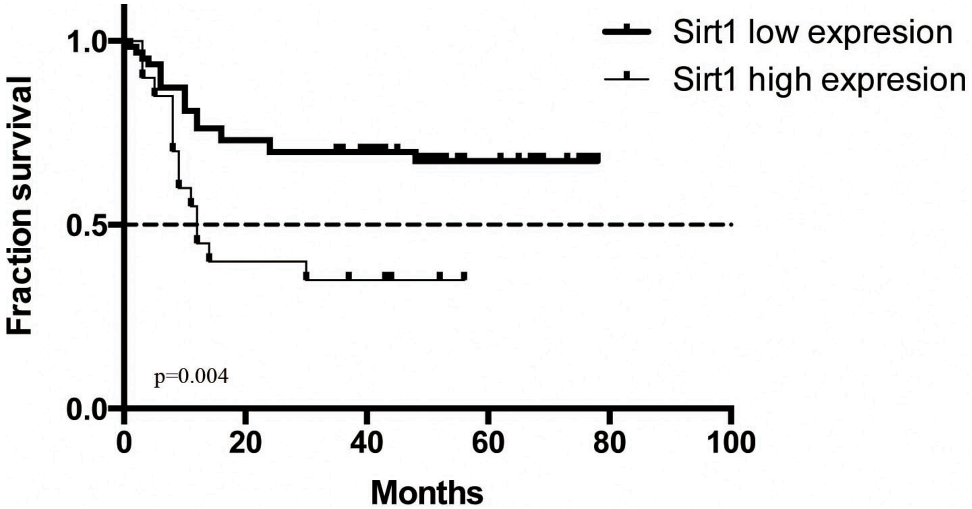

Supplementary Figure 1: Progression-free survival curves demonstrating the relationship between survival prognosis of gastric cancer patients and gene expression. (high SIRT1 expression vs. low SIRT1 expression in advanced gastric cancer patients).

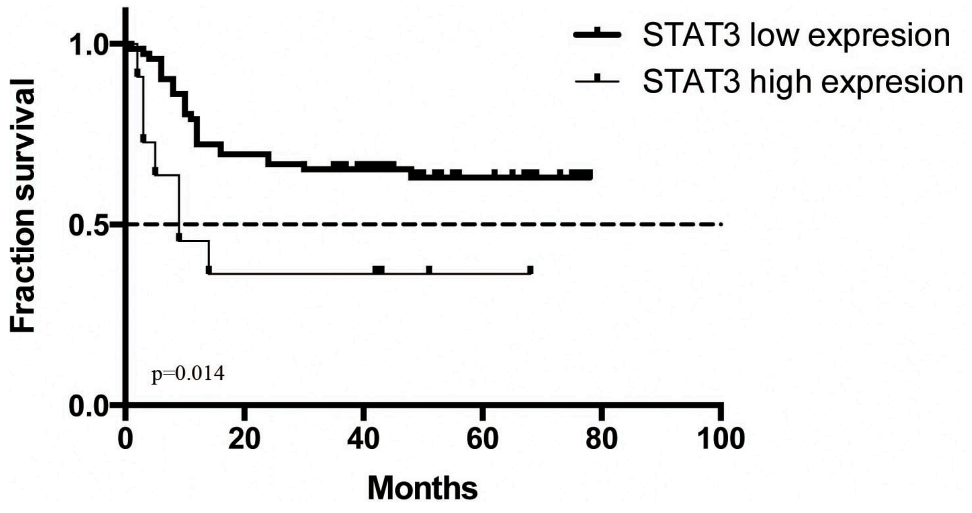

Supplementary Figure 2: Progression-free survival curves demonstrating the relationship between survival prognosis of gastric cancer patients and gene expression. (high STAT3 expression vs. low STAT3 expression in advanced gastric cancer patients).

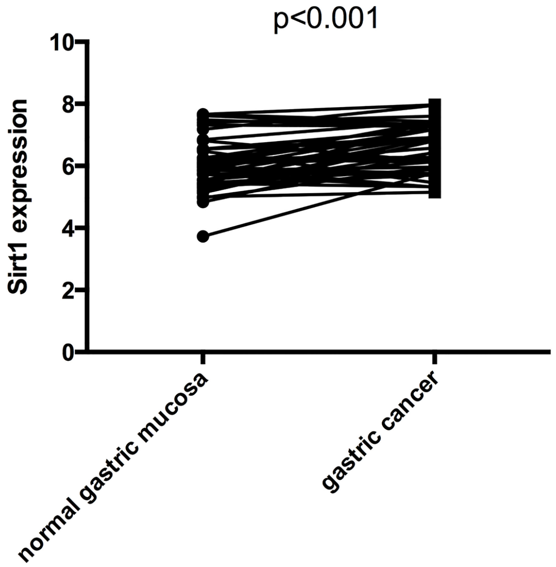

Supplementary Figure 3: Differential mRNA expression of SIRT1 in gastric cancer tissues from patients in GSE63089 dataset compared to their matched normal gastric mucosa (values expressed as Mean ± SEM).

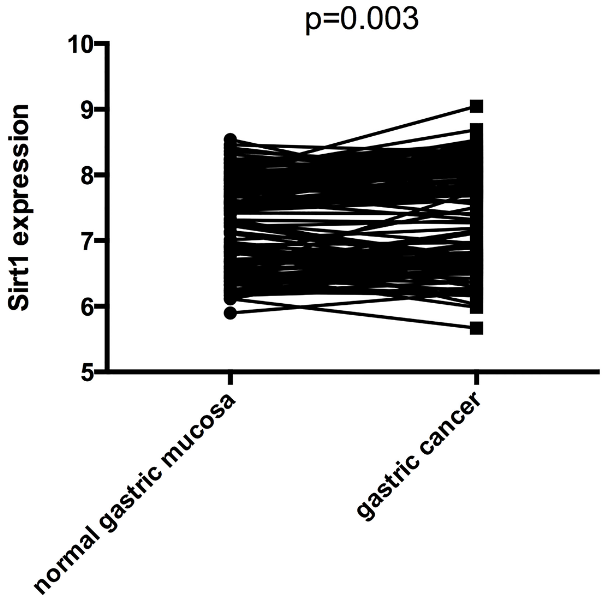

Supplementary Figure 4: Differential mRNA expression of SIRT1 in gastric cancer tissues from patients in GSE29272 dataset compared to their matched normal gastric mucosa (values expressed as Mean ± SEM).

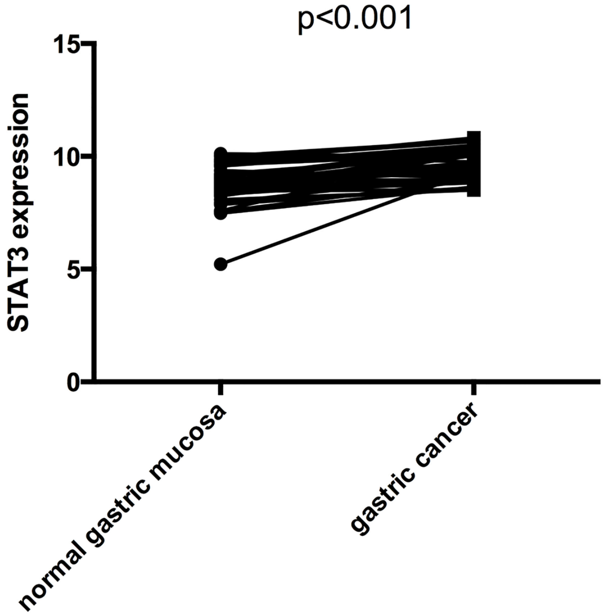

Supplementary Figure 5: Differential mRNA expression of STAT3 in gastric cancer tissues from patients in GSE63089 dataset compared to their matched normal gastric mucosa (values expressed as Mean ± SEM).

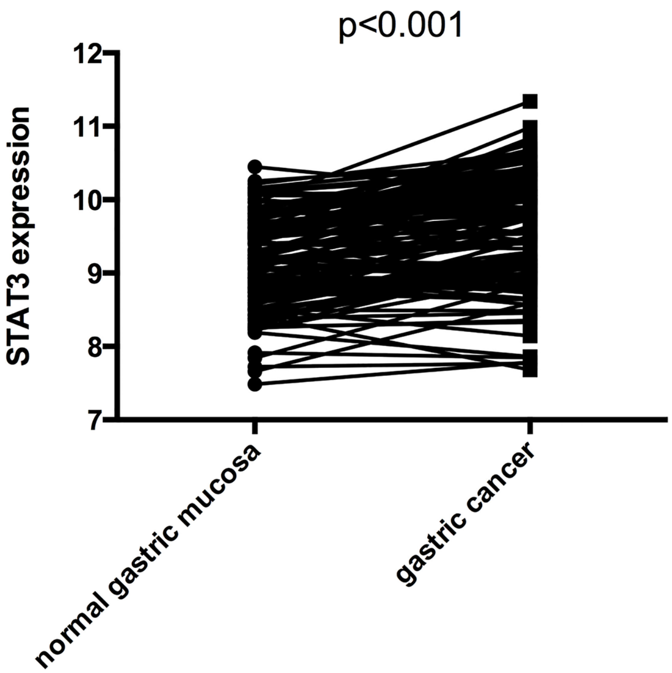

Supplementary Figure 6: Differential mRNA expression of STAT3 in gastric cancer tissues from patients in GSE29272 dataset compared to their matched normal gastric mucosa (values expressed as Mean ± SEM).

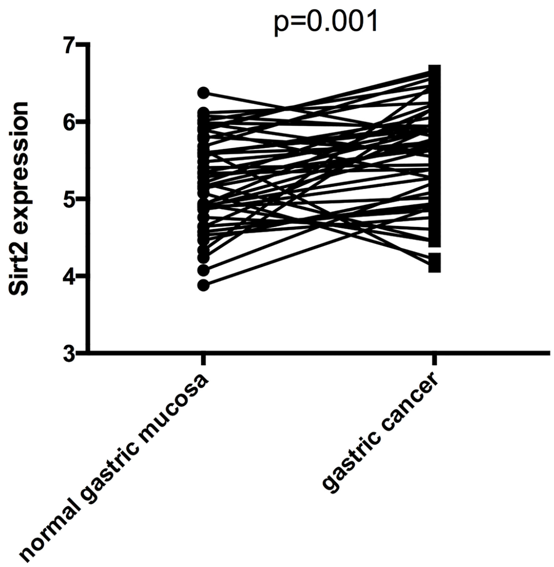

Supplementary Figure 7: Differential mRNA expression of SIRT2 in gastric cancer tissues from patients in GSE63089 dataset compared to their matched normal gastric mucosa (values expressed as Mean ± SEM).

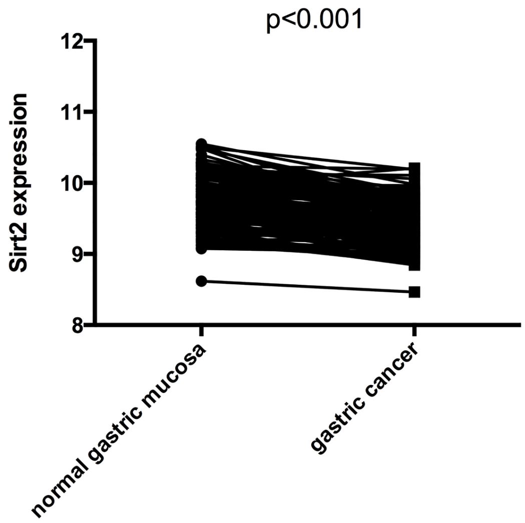

Supplementary Figure 8: Differential mRNA expression of SIRT2 in gastric cancer tissues from patients in GSE29272 dataset compared to their matched normal gastric mucosa (values expressed as Mean ± SEM).
